# Finite element analysis of internal fixation with clavicular hook plate vs. adjustable loop plate for Neer Type IIB distal clavicle fractures

**DOI:** 10.3389/fsurg.2026.1806843

**Published:** 2026-04-13

**Authors:** Liwen Zheng, Baopeng Li, Xi Wang

**Affiliations:** Chengdu BOE Hospital, Chengdu, China

**Keywords:** distal clavicle fracture, Neer IIB Type, clavicular hook plate, adjustable loop plate, finite element analysis, biomechanics

## Abstract

**Objective:**

To compare the biomechanical stability of clavicular hook plates vs. adjustable strap plates in treating Neer Type IIB distal clavicle fractures, providing evidence for clinical procedure selection.

**Methods:**

Based on CT data from a healthy volunteer, a three-dimensional finite element model of the shoulder joint—including bones, ligaments, and internal implants—was constructed using Mimics, Geomagic, and SOLIDWORKS software. Model A simulated fixation with a clavicular hook plate, while Model B simulated fixation with an adjustable strap plate. Muscle loads (simulating holding a 0.5 kg teacup) and boundary conditions were set based on literature. Static mechanical analysis was performed in ANSYS Workbench. Observed metrics included Total Deformation at the path node on the clavicular upper surface (assessing stability) and Equivalent Stress (assessing refracture risk), with statistical comparisons conducted.

**Results:**

Under simulated loading, both models provided effective fixation. Internal fixation material Stresses remained below their respective yield strengths (titanium alloy: 830 MPa; suture: 1,208.87 ± 164.87 MPa), indicating no fracture risk. Model A (hook plate) exhibited a smaller maximum Deformation on the clavicle (0.54 mm) compared to Model B (0.79 mm), but the mean Deformation at path nodes showed no statistically significant difference (*P* > 0.05). The mean Equivalent Stress at the clavicular path node for Model A (3.42 MPa) was greater than that for Model B (2.87 MPa), with a statistically significant difference (*P* < 0.05). The maximum Stress in Model A was concentrated at the hook bend (221.15 MPa), potentially generating localized high pressure on the subacromial surface; the maximum Stress in Model B was located at the midpoint of the strap connection line (278.67 MPa).

**Conclusion:**

Finite element analysis indicates that both the clavicular hook plate and the adjustable strap plate provide adequate biomechanical stability for Neer Type IIB distal clavicle fractures. While both demonstrate comparable fixation strength, they exhibit distinct Stress distribution characteristics. Considering the risk of complications such as subacromial impingement associated with clavicle hook plates, while arthroscopic strap plate techniques can simultaneously address concomitant shoulder injuries without requiring secondary surgery for removal, the latter may offer greater overall advantages.

## 1 Introduction

Fractures of the lateral third of the clavicle are typically classified using the Neer classification system. In Neer Type IIB fractures, the trapezoid ligament remains intact while the conoid ligament is ruptured, resulting in poor fracture stability. Due to factors such as arm weight, scapular rotation, and muscle traction (e.g., pectoralis major, latissimus dorsi, trapezius), delayed union and nonunion occur at a relatively high rate. Early surgical intervention is generally recommended (1). Internal fixation with a clavicular hook plate is currently a common surgical approach for treating distal clavicle fractures, offering good stabilization and a high fracture healing rate (2). However, the hook plate invades the subacromial space and acromioclavicular joint, potentially leading to complications such as acromioclavicular joint osteoarthritis, rotator cuff injury, subacromial impingement, and osteolysis (3). Studies indicate that the overall complication rate following clavicle hook plate fixation can reach as high as 76.6% (4). Therefore, regardless of complication occurrence, it is generally recommended that implants be removed promptly after fracture healing (5). In recent years, there has been increasing research on the use of arthroscopic sling plate fixation for treating distal clavicle Neer IIB fractures. This approach not only reduces the incidence of the aforementioned complications (6), but also allows for simultaneous management of associated injuries such as rotator cuff tears, glenoid labrum tears, and biceps tendon injuries during surgery (7). While numerous case reports and surgical techniques for both approaches exist, studies evaluating their postoperative biomechanical performance remain scarce. This study compares the biomechanical stability of hook plates vs. adjustable strap plates using finite element analysis, aiming to provide biomechanical evidence for clinicians to rationally select treatment modalities.

## 2 Materials and methods

### 2.1 Construction of three-dimensional geometric models of the shoulder joint

**Figure 1:**
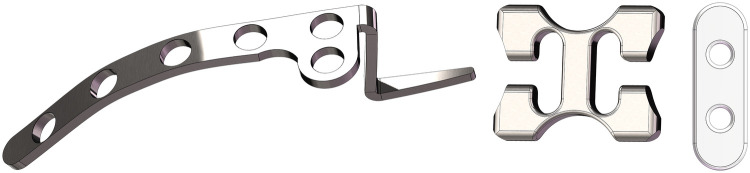
Component.

**Figure 2:**
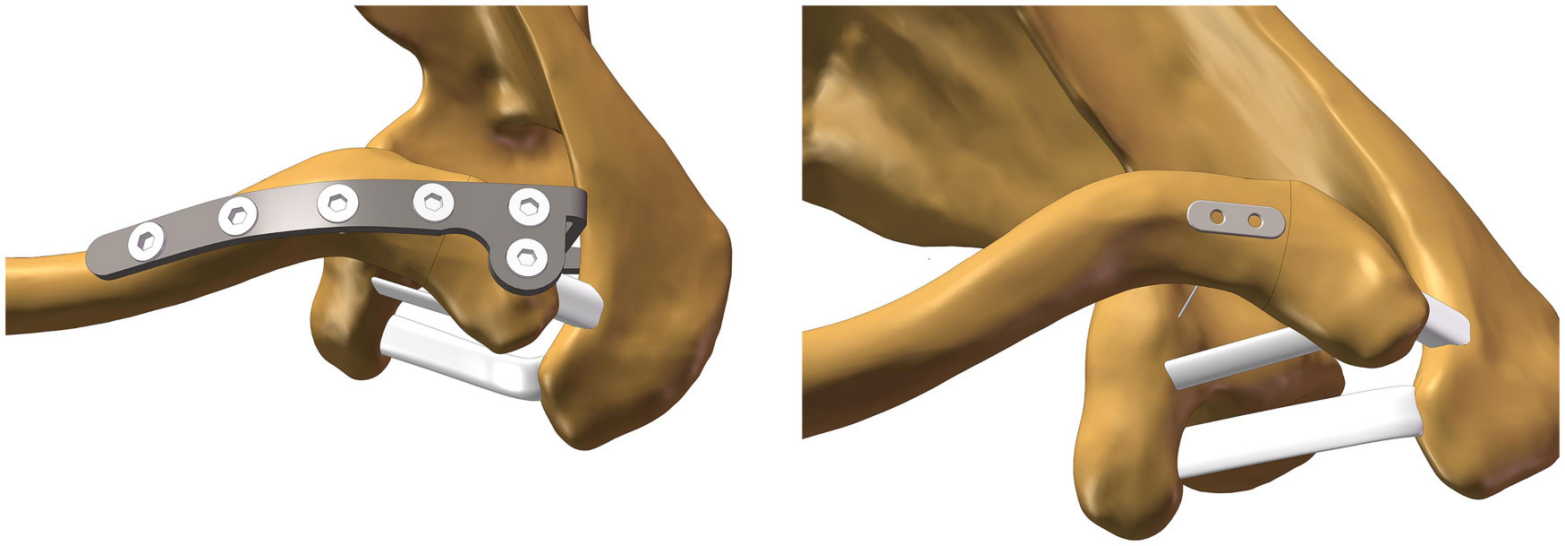
Model A and Model B.

A healthy 28-year-old female volunteer with a height of 165 cm and a weight of 58 kg, and no history of shoulder trauma or developmental abnormalities, was enrolled in this study. This study was approved by the hospital ethics committee prior to commencement, and informed consent was obtained from the volunteer. A 64-slice 128-layer spiral CT scan was performed on the left shoulder joint (1 mm slice thickness). The raw DICOM files were imported into Mimics Research 21.0 software. Defects were repaired using masking, region growing, and brush filling operations to generate solid models of the clavicle and scapula. These models were exported in STL format. The STL files were imported into Geomagic Wrap 2021 for 3D data processing. Operations including remeshing, boundary and spike removal, feature elimination, precise surface refinement, and surface fitting were performed sequentially to generate a 3D shoulder joint model (STEP format). Using SOLIDWORKS 2023 software and adhering to national medical device standards (YZB/National 3883-2013, YY0018-2016) to create sheet metal components including clavicle hook plates, screws, sling plates, and cloverleaf plates (Figure 1). Import the STEP file into SOLIDWORKS 2023 and assemble it according to model requirements into a clavicle hook plate assembly (Model A) and a sling plate assembly (Model B). Based on actual anatomical relationships, the acromioclavicular ligament, coracoclavicular ligament, and trapezoid ligament were added. Add a reference plane perpendicular to the clavicle at its distal end to divide the clavicle, simulating a Neer IIB fracture (Figure 2). The trapezoid ligament is preserved at the fracture site, while the conoid ligament is removed.Following the remodel, the fracture plane is positioned between the trapezoid ligament and the conoid ligament, with the fracture line located lateral to the conoid ligament. The precise location is 18.76 mm medial to the distal articular surface of the clavicle. The suture cord used during surgery consists of two strands. Since both strands pass through the same bone tunnel, it can be simplified to a single strand to reduce computational complexity. The suture employed is a size No. 2 suture cord with a diameter of 0.4 mm.After verifying no interference existed in all models, the assemblies were finally exported in x_t format.

### 2.2 Finite element analysis model construction

The x_t format file was imported into the finite element analysis software Workbench 2022 R1. Various titanium plates, bone tissue, and plate-to-plate connection wires were modeled as homogeneous, isotropic linear elastic materials, assigned corresponding material parameters (8–10). Ligaments in non-osseous tissues exhibit strong nonlinear and anisotropic properties (11). Simulating them with incompressible springs is inaccurate; such hyperelastic materials are typically described using the Neo-Hookean simplified model (12) (Table 1). Contact relationships were added based on the software's automatic contact detection, with friction relationships set between bone-bone and suture-bone interfaces, using friction coefficients of 0.4 (13) (Table 2). Meshing was performed for both models with an element size of 1 mm. After meshing, Model A contained 49,274 elements and 84,596 nodes, while Model B contained 85,658 elements and 26,395 nodes. During the solution process, both models converged, enabling the calculation of Equivalent Stress and Total Deformation at various locations.

**Table 1:** Material parameters for each component.

Linear elastic material		
Component	Young's modulus[MPa]	Poisson ratio
Cortical bone	11,000	0.3
Titanium plate(Ti-6Al-4 V)	1,14,000	0.34
FiberWire® 2 Suture	10,729.4	0.4
Superelastic material		
Component	Initial shear modulus[MPa]	Incompressibility parameter
Ligament	1.44	0.00126

**Table 2:** Relationships among contact surfaces.

Contact surface	Contact relationship
Bone and screw, screw and steel plate, bone and steel plate	Binding
Bone to bone, bone to suture	Friction (coefficient of friction 0.4)

### 2.3 Boundary conditions and load setup

**Figure 3:**
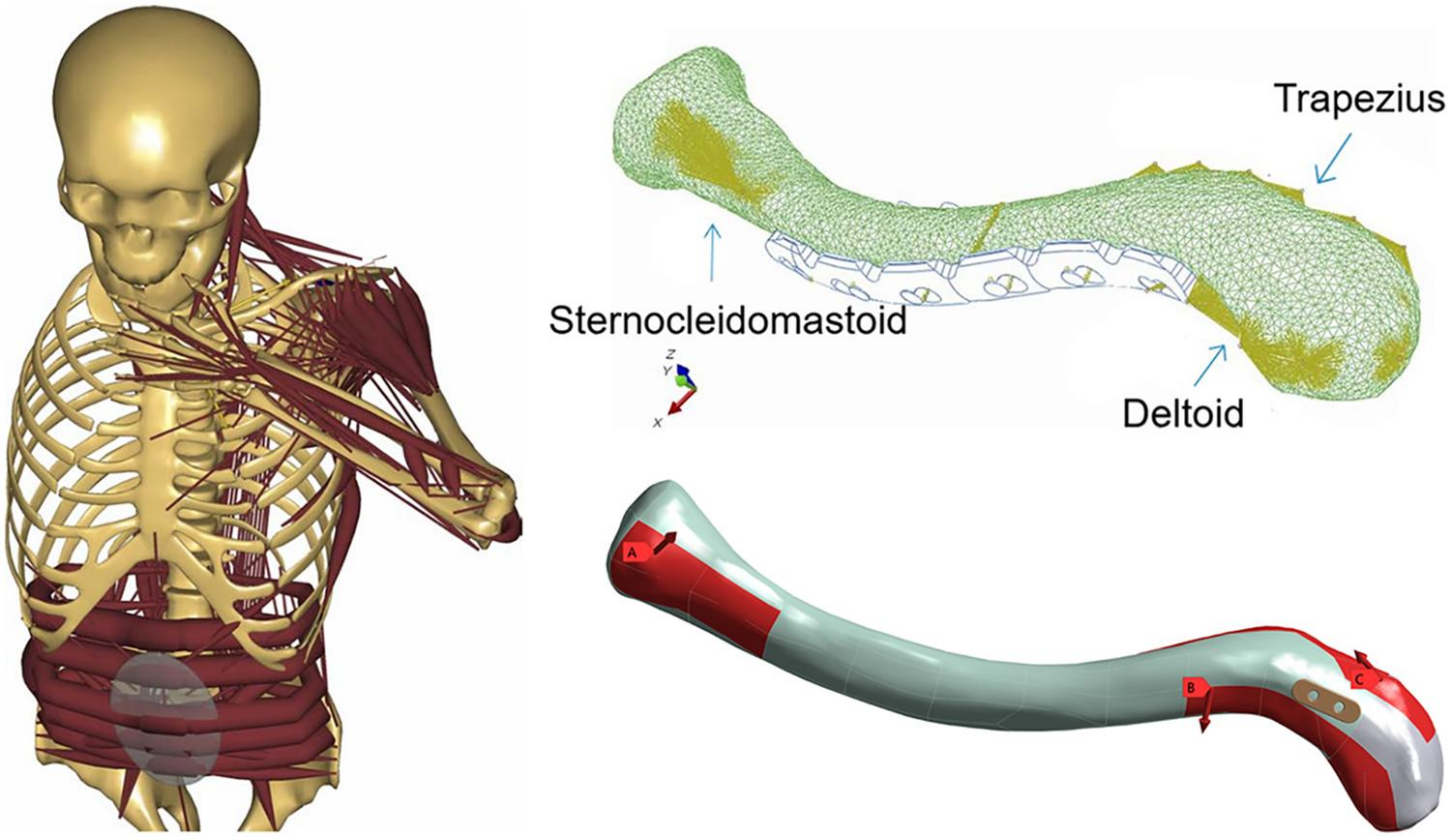
Load diagram.

Load conditions were selected based on research by Cronskär et al. (14), representing a static posture where one arm holds a 0.5 kg teacup in front of the mouth. Load application points and magnitudes for the clavicle were added (Table 3, Figure 3). Boundary conditions: The proximal sternoclavicular joint surface and the undersurface of the acromion were fixed, defining the positions of these two surfaces (i.e., their Deformation along the X, Y, and Z axes were set to zero). The solver was instructed to enable large deflection in the static structure.

**Table 3:** Muscle forces on the clavicle from the multibody musculoskeletal simulation[N].

Muscle	Fx	Fy	Fz
Deltoid	59.2	−46.8	−25.8
Sternocleidomastoid	−4.2	14.2	−1.5
Trapezius	−2.8	22.4	30.5

**Table 4:** Comparison of data from this study and other studies.

Group	Stress range of steel plates [Mpa]	Maximum stress in steel plate [Mpa]	Maximum stress on the bone [Mpa]	Maximum skeletal deformation [mm]
This study	42–177	221	8.6	0.54
Other studies	70–140	171	58	0.25

### 2.4 Observation indicators and statistical methods

The geometric path from proximal to distal on the supraclavicular surface of both model groups was observed. Total Deformation, Equivalent Stress (Von Mises), maximum Deformation, and maximum Equivalent Stress across the entire path and model were recorded. Data were analyzed using IBM SPSS Statistics 22 software. Since the overall data did not follow a normal distribution, quantitative data are expressed as median (Q1, Q3). Intergroup comparisons were performed using the Mann–Whitney *U*-test, with *P* < 0.05 indicating statistically significant differences.

## 3 Results

### 3.1 Model stability assessment

The stability of the clavicle was evaluated by analyzing the Total Deformation of all nodes along the supraclavicular surface path. For Model A, the maximum Deformation occurred at the distal end of the clavicle, with a peak Deformation of 0.54 mm. For Model B, the maximum Deformation occurred at the proximal fracture line, with a peak Deformation of 0.79 mm. Model A exhibited lower Deformation than Model B.

**Figure 4:**
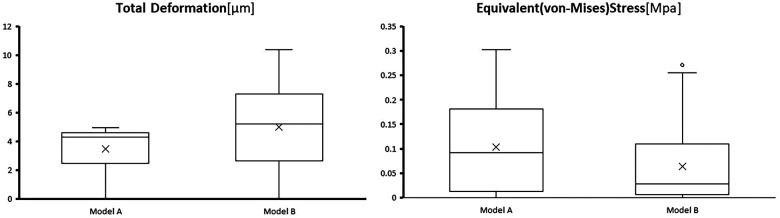
Statistical results for the supraclavicular surface pathway.

**Figure 5:**
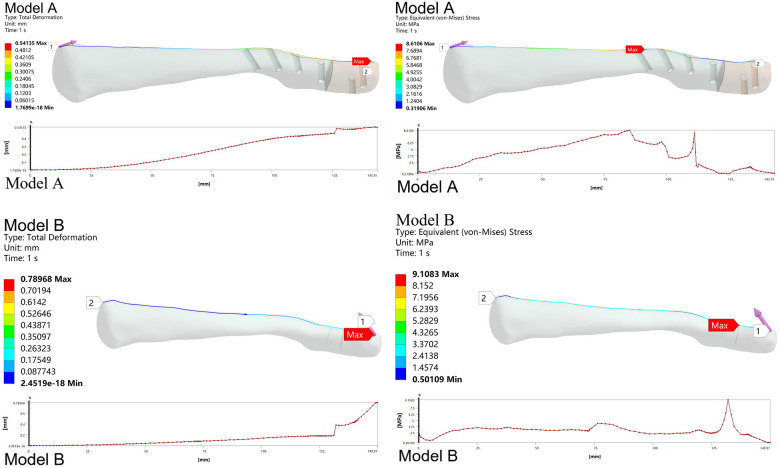
Total deformation and equivalent stress along the supraclavicular surface path.

Across all nodes (124 total), the overall mean Deformation for Model A was 3.42 (1.31, 5.87) mm, while Model B's overall mean Deformation was 2.87 (2.27, 3.25) mm. The difference was not statistically significant (*z* = −1.585, *p* > 0.05) (Figures 4, 5).

**Figure 6:**
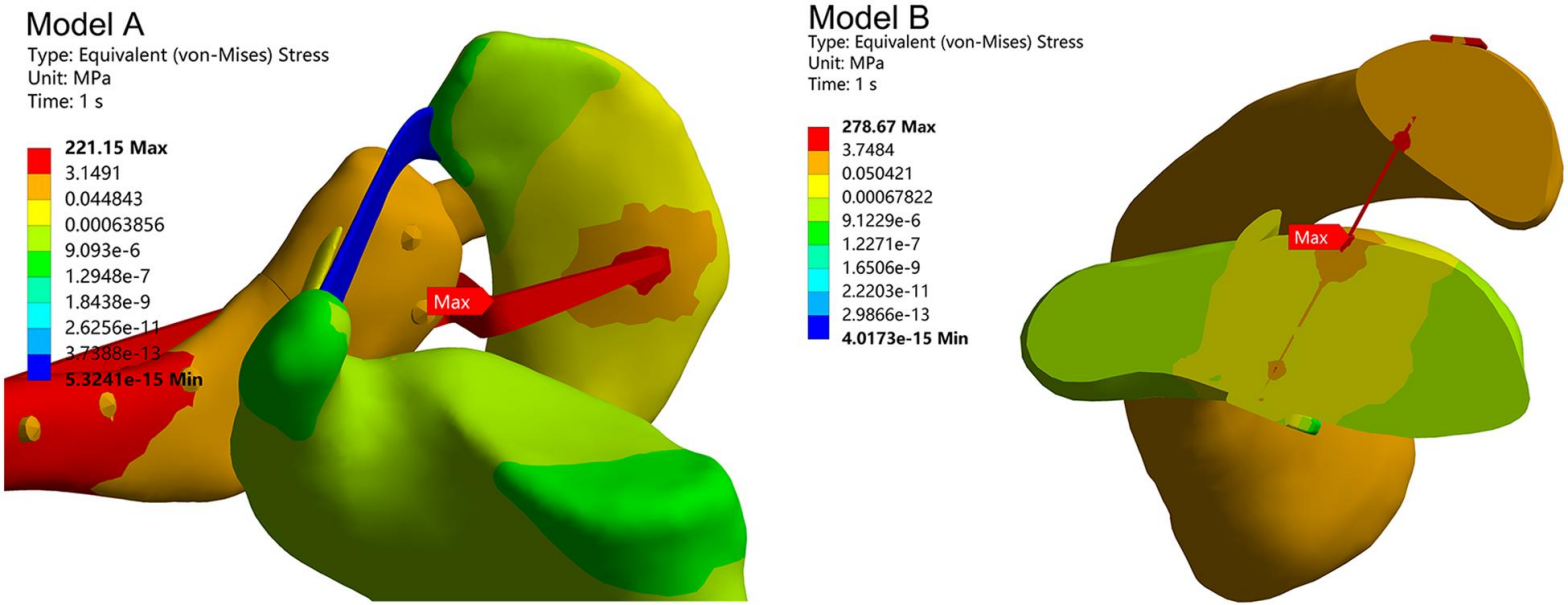
Detail of a section.

Across the entire model, the maximum Deformation in Model A occurred at the distal end of the clavicle, with a peak value of 0.59 mm. The maximum Deformation in Model B occurred at the posterior aspect of the distal clavicle, reaching 1.31 mm (Figure 6).

### 3.2 Model stress evaluation

The risk of clavicle re-fracture was assessed by evaluating the Equivalent Stress at all nodes along the superior clavicular surface path. For Model A, the maximum Equivalent Stress occurred at the most proximal nail hole, reaching 8.61 MPa. For Model B, the maximum Equivalent Stress was observed at the distal clavicle, peaking at 9.11 MPa. The maximum Equivalent Stress was lower in Model A than in Model B.

**Figure 7:**
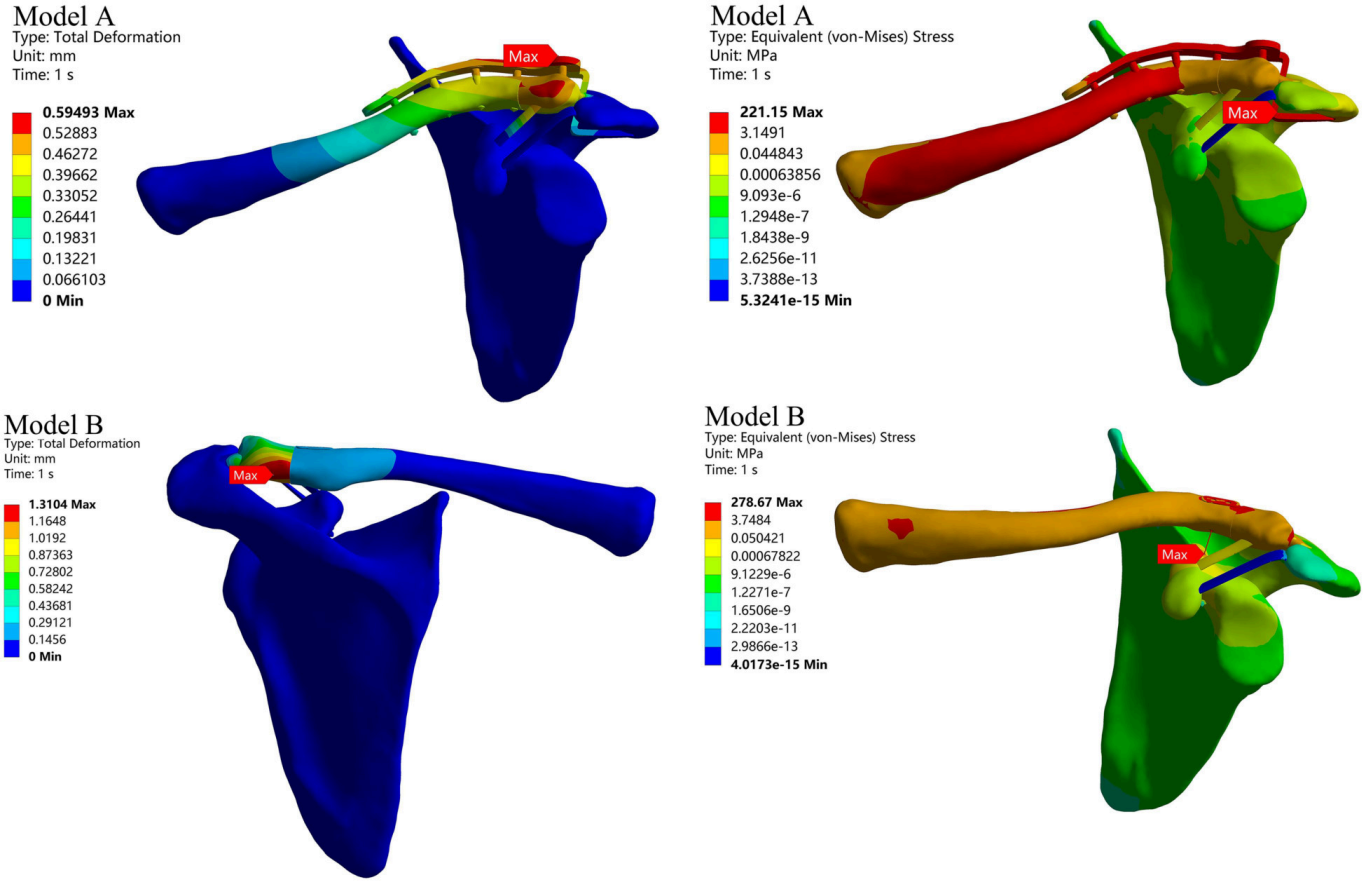
Total deformation and equivalent stress for the entire model.

Across all nodes (124 total) along the path, the mean Equivalent Stress for Model A was 3.42 (1.31, 5.82) MPa, while Model B's mean Equivalent Stress was 2.87 (2.27, 3.25) MPa. Model A exhibited a higher mean Stress than Model B. The difference was statistically significant (*z* = −3.593, *p* < 0.05) (Figures 4, 7).

Throughout the entire model, the maximum Equivalent Stress in Model A occurred at the bend of the hook plate clavicle hook, reaching 221.15 MPa. The maximum Equivalent Stress in Model B occurred in the middle section of the sling connection line, reaching 278.67 MPa (Figure 6).

### 3.3 Model validation

Compared with previous studies, this research shows no significant differences in Stress range, maximum Stress, and bone Deformation. Therefore, the model is deemed suitable for finite element simulation of the shoulder joint (15) (Table 4).

## 4 Discussion

The clavicle hook plate is a classic treatment for distal clavicle fractures. The plate not only stabilizes the fracture fragments but also establishes a connection between the entire clavicle and the acromion via the clavicle hook, enhancing distal clavicle stability. Its fixation strength is unquestionable. In this experiment, the Total Deformation of the fracture fragments in Model A was smaller than that in Model B, corroborating this view. In the Stress distribution diagram, the maximum Stress in Model A occurred at the bend of the clavicle hook, with an Equivalent Stress of 221.15 MPa. This value is lower than the yield strength of 830 MPa (10), indicating that under this loading condition, there is no risk of plate fracture. At the contact zone between the acromion and clavicular hook (Figure 6), the applied load transmits through the plate, generating localized peak pressure on the undersurface of the acromion. This scenario may induce subacromial pain or osteolysis, with incidence rates as high as 37.7% reported in Joo's study (16). In the Total Deformation diagram, both Model A and Model B exhibited maximum Deformation at the distal clavicle, confirming that Neer Type IIB fractures are unstable. Early upper limb mobilization should be avoided postoperatively.

The coracoclavicular ligament is a crucial stabilizing structure at the distal clavicle. Rupture of the conoid ligament within this complex is a primary factor in fracture Deformation. Therefore, conoid ligament reconstruction is vital for achieving and maintaining fracture reduction (3). The clavicle hook plate addresses coracoclavicular ligament injuries by stabilizing the entire shoulder joint, providing a mechanically stable environment for ligament repair. However, the elastic modulus of titanium alloy is over ten times that of cortical bone. Excessively rigid fixation often leads to osteoporosis and Stress shielding, resulting in low local bone strength and a high risk of re-fracture even after internal fixation removal. The sling plate technique nearly achieves *in situ* fixation of the clavicle to the coracoid process via the coracoclavicular ligament, similarly providing a stable healing environment between the acromioclavicular and coracoclavicular joints (17). In Model B group, the maximum Deformation also occurred near the anatomical position of the coracoid ligament, but the Deformation magnitude was smaller. This aligns with the recently proposed biomechanical concept of elastic fixation. Research by Gan K et al. (18) suggests that elastic fixation can achieve equivalent clinical outcomes to static fixation while eliminating the risk of reoperation for internal fixation removal. In the Stress distribution diagram of Model B, the maximum Stress occurred in the middle section of the sling (Figure 6), with a value of 287.67 MPa. This is lower than the highest fracture Stress value reported (9) (1,208.87 ± 164.87 MPa), indicating no risk of sling fracture under this loading condition. Most distal clavicle fractures occur following falls with direct shoulder impact, while a minority result from falls with hand extension. In Marin et al.'s (7) study, 17.70% of acute distal clavicle fracture patients presented with concomitant glenohumeral joint injuries, with rotator cuff tears, glenoid labrum tears, and bicipital pulley lesions being the most common associated pathologies. 84.21% of cases required additional surgical intervention. The arthroscopic approach with a plate with a band can simultaneously address such injuries.

In this study, even though the Deformation in both model groups was comparable and at the millimeter level, it did not cause fracture Deformation. Moderate micro-movement of the fracture could even stimulate fracture healing (19). The Stress in all models was below the material yield strength, and the internal fixation was stable. Therefore, both methods provide adequate fixation for distal clavicle fractures. However, in terms of complication management and associated injury treatment strategies, the suture-anchor plate offers advantages over the hook plate approach.

## Data Availability

The raw data supporting the conclusions of this article will be made available by the authors, without undue reservation.
